# Complex mitral valve anatomy and open issues in transcatheter mitral valve replacement

**DOI:** 10.1016/j.sipas.2023.100182

**Published:** 2023-05-25

**Authors:** Haroon Zafar, Sajjad Soleimani, Masooma Ijaz, Junaid Zafar, Faisal Sharif

**Affiliations:** a Cardiovascular Research & Innovation, School of Medicine, University of Galway, Galway, Ireland; b Lambe Institute for Translational Research, University of Galway, Galway, Ireland; c College of Science and Engineering, University of Galway, Galway, Ireland; d Department of Chemistry, Materials, and Chemical Engineering, Politecnico di Milano, Milan, Italy; e Faculty of Engineering, Government College University, Lahore, Pakistan; f Department of Cardiology, University Hospital Galway, Galway, Ireland; g CÚRAM-SFI Centre for Research in Medical Devices, Galway, Ireland

**Keywords:** Mitral valve, Trans-septal puncture, Transcatheter

## Abstract

Higher and prohibitive mitral valve disease surgical scenarios are preferred cases for transcatheter mitral valve replacement as they offer unrelenting mitral valve regurgitation reduction. This review entails medical technologies that are evolving bioprosthetic devices for mitral valve repair and replacement purposes. Transcatheter mitral valve replacement is compared with transcatheter aortic valve implantation based on the etiology and driving factors. Leading anchoring systems to place and fix the mitral valve prosthesis in left atrium/ventricle annulus are discussed. Furthermore, accessing modalities to stretch to the mitral valve including transapical, trans- aorta and transseptal are included along with the associated key challenges.

## 1 Introduction

**Fig. 1:**
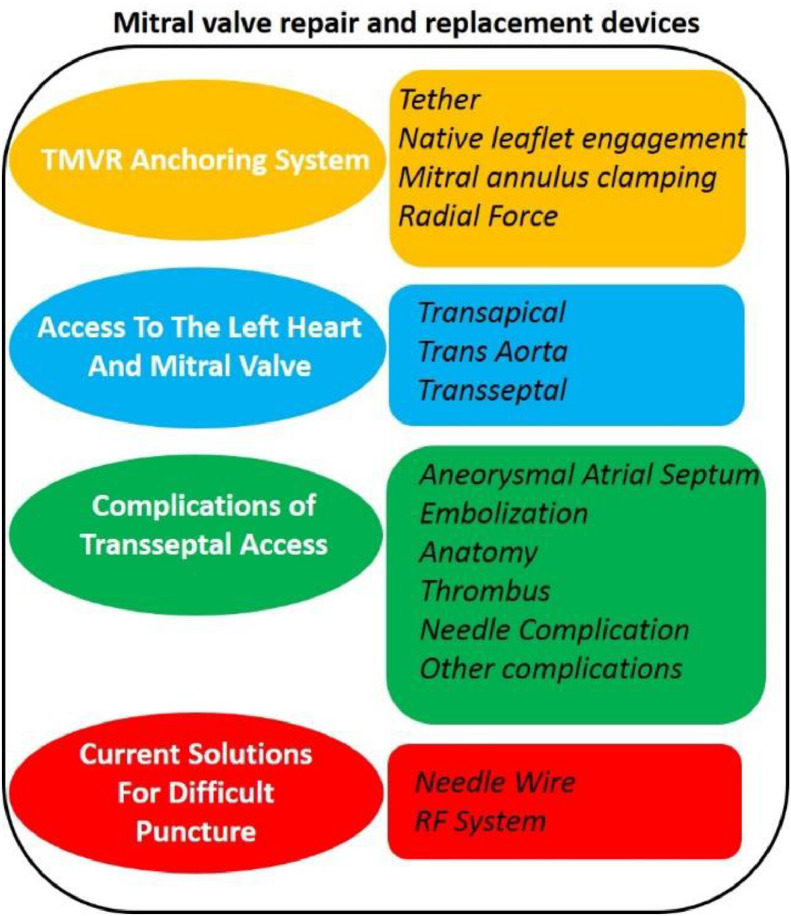
Mitral valve repair and replacement devices under FDA trial studies.

It is established that transcatheter mitral valve replacement (TMVR) is a complex procedure due to the mitral valve (MV) anatomy, shape and its inter- dependencies on nearby structures. To evolve an improved TMVR solution relevant pre-procedural analysis is incumbent to examine mitral regurgitation (MR), subject suitability as per anatomic and implementation characteristics. Important considerations must include MV orientation, closeness of the left ventricular outflow tract, thrombogenicity and anchoring apparatus [1], [2], [3], [4]. This review aims to pitch on the available devices in MV repair and replacement and their potential complications. More than 30 companies are developing bioprosthetic devices for MV replacement with almost similar number trying to evolve devices MV repair. Among all these efforts, only Mitraclip from Abbott [5] has FDA approval for degenerative MR treatment. Regarding the EU market, only the products received CE market approval so far are Carillon, Mitralign, Neochord and PASCAL. The devices under FDA trial studies are summarised in Fig. 1.

The devices under FDA trial studies are (a) Carillon from Cardiac Dimension uses the anchor and a shaping ribbon to seal expanded mitral annulus in secondary MR, (b) Cardioband from Edwards lifesciences uses semirigid, posterior annuloplasty ring, and (c) Neochord DS100 from Neochord) uses sutures as artificial chords in degenerative MR.

Some other devices depending on the target (leaflet, chords, annulus) for MV repair are; (a) Targeting Leaflet, (b) Pascal (Edwards), (c) Mitraflex (transcardiac), and (d) Pascal and MitraFlex which use spacer or paddle to overcome MR. Some Targeting Chords are (a) Neochord which has CE market since 2012 and is under FDA trial RECHORD and (b) Harpoon which is under CE TRACER trial. Cardioband and Amend directly remodel annulus. Carillon, and ARTO which indirectly shape the annulus to overcome MR.

It should be noted that there is an increasing intendancy toward whole MV replacement in recent years. However, there are several difficulties remain as of technology barrier when the TMVR is compared with Transcatheter Aortic Valve Implantation (TAVI). The following are the most important difficulties to be challenged [6].

a ***Large annulus****:* large annulus needs a larger delivery device, which causes bigger incision or puncture depending on the accessing method.
b ***D-shaped annulus****:* the asymmetrical shape of the annulus may be the cause of paravalvular leakage (PVL) specifically in case of deployment of symmetrical prosthesis. It may also lead to interfering with adjacent structure to mitral annulus if a symmetrical prosthesis is deployed. There are few mitral valve prosthesis devices which have a D-shaped structure (such as TIARA). The deployment of such devices however needs extra effort and care.
c ***Lack of a fibrous/calcified annular structure:*** Lack of calcified structure on the annulus (in Functional MR) is a downside aspect as it does not provide a firm structure to anchor and fix the implanted prosthesis at the proper location on mitral annulus to prevent further migration, which may lead to a new PVL.
d ***Complex subvalvular apparatus:*** The complex subvalvular apparatus should be treated with extra care during the process of accessing to MV as well as the implantation of device. Any unmanaged interfering to the subvalvular apparatus may lead to left ventricular (LV) deformation or left ventricular out flow tract obstruction (LVOTO).
e ***Proximity of the mitral valve to adjacent important structures:*** The proximity of MV to left ventricular out flow tract, circumflex coronary artery and coronary sinus (CS) may be problematic if the prosthesis or process of prosthesis deployment interfere with their function.

Regarding the prosthesis devices for TMVR, currently there are several devices under early feasibility or trial study. The devices dedicated to MR are made with NiTi and self-expandable. Often the device to treat mitral valve stenosis (MS) are balloon expandable (such as Edward SAPIEN) and could be off the shelf used on aortic transcatheter heart valve such as LOTUS (Boston Scientific). Majority of these devices are equipped with three leaflets Glutaraldehyde fixed porcine pericardium. They exploit, however, various anchoring mechanism, delivery system and accessing method to the left heart. The followings indicate some of those bioprosthesis devices, which are under trial studies:

a ***Transseptal/Transapical (TS/TA) access:*** RELIEF trial study is for CardiaAQ-Edwards™ transcatheter mitral valve (Edwards Lifesciences; Irvine, CA), SITRAL study is for Edwards SAPIEN 3 transcatheter heart valve (Edwards Lifesciences; Irvine, CA), MITRAL study is for Edwards SAPIEN XT and SAPIEN 3 transcatheter heart valve (Edwards Lifesciences; Irvine, CA).
b ***TS access:*** PRELUDE study is for Caisson transcatheter MVR system (Caisson Interventional LLC; Maple Grove, MN)
c ***TA access:*** TIARA-II Tiara™ (TMVR Study) for Tiara™ valve and transapical delivery system transcatheter MVR (Neovasc Inc; Richmond, B.C. Canada), Expanded Clinical Study of Tendyne Mitral Valve System study for Tendyne™ Mitral Valve System (Tendyne Holdings, LLC, a subsidiary of Abbott Vascular, Roseville, MN) [7].

## 2 TMVR anchoring system

The widely various and creative anchoring systems to place and fix the mitral valve prosthesis in left atrium (LA)/left ventricle (LV) annulus along with their pros and cons are provided below and discussed briefly [8].

***Tether:*** The bioprosthesis device is secured at its location at LA/LV **t**hrough the axial tension provided by the tether. The tether connects the bottom part of the MV bioprosthesis to the LV apex. As long as remodeling is not occurring on the left heart, the bioprosthesis device might fit at its location and properly seals any PVL; however, in case of remodeling, the tether may become too loose or too tight and cause PVL or leads to the dysfunction of structures adjacent to the MV annulus. Examples of such devices are Tendyne valve- Tendyne Medical Inc., Baltimore, Maryland, US, Mitral Seal-Avalon Medical Ltd.
***Native leaflet engagement:*** The bio-prosthesis have the anchors to grasp the natural leaflet in order to be secured at its position. Implantation of device could be challenging, as during the device release from the sheath, it needs a proper orientation. Moreover, the device securing may depend on the leaflet, and in case of calcification, the leaflet may not be able to provide a proper anchoring basis. However, once the proper anchoring achieved, the risk of LVOTO will significantly diminish as the anterior leaflet are not free to obstruct the out-flow tract. Examples of such devices are FORTIS, Edwards Lifesciences, TIARA, NeoVasc Inc., and MITRASSIST-MitrAssist Medical Ltd.
***Mitral annulus clamping:*** The bioprosthseis device will be secured at LA/LV annulus through squeezing the annulus, which could be enhanced by a ring. Such squeezing may increase the LVOTO risk or interfere with the adjacent structure. The examples of such devices are DOUBLE-CROWNED Lausanne & Zhejiang Uni. and GORMAN University of Pennsylvania.
***Radial force:*** The bioprosthesis is secured at its position through radial force. Radial force may not be favored as it can interfere with the function of adjacent structures such as CS and increase the risk of LVOTO. Examples of such devices are Intrepid Valve, Medtronic, Minneapolis, Minnesota.

## 3 Access to the left heart and mitral valve

There are various access methods to reach the MV. Some of these methods are describes as follows [8].

***Transapical:*** TA approach is the most applied method by several medical device producer. TA method provides an easy access to the MV annulus with great coaxially to deploy the MV prosthesis. However, it is more invasive than other methods. It also navigates through subvalvular apparatus and will have negative effect at LV myocardium. This issue may negatively affect the cardiac output in a population who mostly already suffer from heart issue including LV myocardium weakness. Examples of such devices are TIARA, Tendyne and FORTIS.
***Trans aorta:*** Trans aorta if is performed through femoral artery would be the least invasive method to access the MV annulus. However, this method engages with several difficulties such as deployment of the device through several curvature such as ascending aorta and the curvature of LV toward the LA. Moreover, there is a risk of subvalvular apparatus interfering as well as presence of calcification at the aortic root that causes the access to the left heart even harder. Since femoral artery supports an access of smaller profile device, an alternative approach could be accessing the device through femoral vein and switching to artery through Trans-Caval (TC) approach by introducing a snare system through femoral artery.
***Transseptal:*** TS approach is the most favoured one to access the mitral annulus for the MV prosthesis delivery. It is less invasive than transapical approach performed percutaneously and minimizes the subvalvular manipulation (although in case of flossing or derailing method there is some risk of interfering with subvalvular apparatus). TS method also avoids LV myocardium weakening as well as complications associated with the thin tissue in LV wall function. However, it should be considered that the TS access to the left heart for current interventions such as atrial fibrillation ablation, left atrial appendage occlusions, Mitraclip procedure and etc., recruit devices with the size of 8 ∼ 22 Fr, while the MV replacement prosthesis delivery devices of current technology has the diameter of 30 ∼ 40 Fr. Further technology with lower profile and proper occlusion method for iatrogenic intra atrial septum defect may benefit TS method and highlights several advantages of TS. It could be expected that the MV replacement technology will be performed through TS method in future. Examples of such devices are CardiaQ and SAPIEN series.

## 4 Complications of transseptal access

**Fig. 2:**
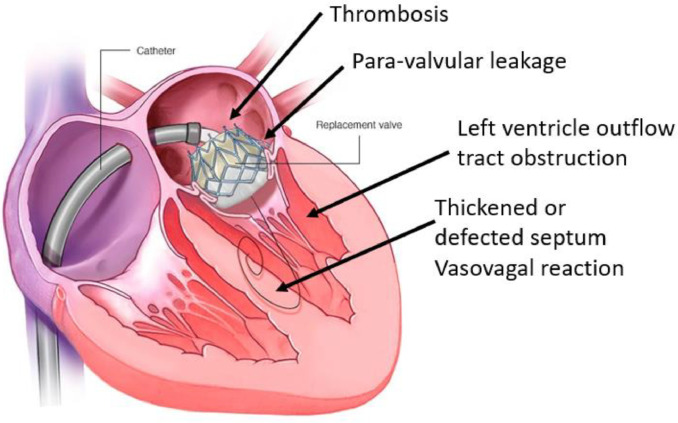
Common complications in transcatheter mitral valve implantation.

Transseptal access possess some complications despite the advancements in surgical advancements in procedures and equipment. These complications are explained as follows. Fig. 2 provides a summary of the current open issues for TAVI.

***Thickened or aneorysmal atrial septum:*** Performing puncture on a thickened septum is much harder than a normal septum. The following patients may develop thickened atrial septum: patient who has performed open heart surgery (FO is sutured), prior valve surgery, atrial septal patching or repair for congenital heart disease and with fibrotic FO (in old patient). Other difficult cases are patients with Aneorysmal Atrial Septum. For such patients the transseptal needle causes "tenting" of the septum. The solution is relied on using extra force, adjusting the needle curvature and the usage of Safesept method. Echocardiography will also help TS puncture in such cases. RF method could be also considerd as an altenative method to needle puncture [9], [10], [11].
***Iatrogenic atrial septum defect (iASD):*** iASD may lead to Hypoxemia, Heart failure and Systemic Embolization. Depending on the size of iASD, it might be essential to close puncture after iASD creation.

The management of iASD is further explained. It should be considered that whether the ASD is associated with any high risk features such as large ASD (> 8 mm), large left to right shunt, right to left shunt with hypoxemia, recurrent DVTs or severe RV dysfunction, History of cryptogenic stroke, Aneurysmal septum, Pacemaker or ICD leads, young age <50, at this situation the closure of ASD should be considered. Otherwise, the patient should undergo 3–6-month surveillance with TEE and TTE. If there is still persistence ASD with some of the high risk factors the ASD closure need to be considered otherwise a follow up of 12 months should be considered which ASD closure should be considered if there will be still persistence ASD even if there is no high risk factors.

As shown through review study by Alkhouli et al., regarding the iASD for 14 and 22 Fr size with 6 and 12 months follow up (moderate to relatively large device), iASD exist even after 6 to 12 months.

Some example of the curent devices to occlude the iASD are GORE Cardioform Septal Occluder (W.L Gore and Associates, Flagstaff, AZ) and AMPLATZER Atrial Septal Occluder (AGA Medical, Plymouth, MN). One problem regarding these devices is at re-puncture making it difficult. An alternative option could be bioresorbable device currently under development such as CARAG septal occluder (CARAG-S Engineering, Baar, Switzerland) [12,13].

TS on patients with repeated Atrial Fibrillation AF is a complication since they may develop septal thickness and distorted anatomy. Up to 40% of patients undergoing AF, may need to repeat the treatment, and 30% of them may develop a difficult case for TS puncture procedure [14].

***Anatomy:*** In pathogonic heart with distorted anatomy the following situation may happen: (a) the LA enlarges, in such cases the puncturing needle should be straightened for an effective puncture, (b) the RA enlarges, at which septum cannot be engaged readily by the tip of the catheter. In such cases, puncturing needle should be curved (greater curvature) for easier engagement with FO, (c) aortic root dilates; in such situation, septum becomes vertical. (d) The other anatomical difficulty is in case of kyphoscoliosis and cardiac rotation [15].
***Thrombus:*** The stainless steel needle is thrombogenic; thus, right after the puncture and entrance to the LA, the heparin is administrated through the catheter/needle holes. It should be noted that the heparin is only administrated after the puncture so that in case of inadvertent perforation the risk of hemorrhage will be prevented (Cardiac tamponade may happen at 0.5∼2% of the cases). Patient with anticoagulant therapy history should be first return to the prothrombin time and then the TS puncture be applied.
***Complication regarding the needle:*** One complication regarding the needle is a choice of wrong needle/sheath curvature. The proper choice of needle curvature will help properly siting of needle on FO and transferring force to it. Other complication regarding the needle is perforation of needle or catheter. In case of needle perforation, the needle should be withdrawan and the whole process should be repeated. It should be noted that needle perfortation is less dangerous if it is not folowed by catheter perforation which should be treated as an emergency case with open heart surgery. Another complication is dilator jump inside the LA and LA wall tamponade, such situation could be avoided with safe sept device or needle wire solution.

If the needle passes the septum but it is not possible to pass the catheter, the operator should use the guide wire as a support, withdrawing the needle and dilating the septum with the balloon. In some situation the whole process should be repeated in a another puncture point at FO [15,16].

***Other complications:*** Vasovagal reaction, which could be due to the pressure on the septum or by mediastinal bruising, atrial arrhythmias due to the presence of the catheter/needle in the RA or LA, pleuritic chest pain, stroke/transient ischaemic attack, transient ST elevation of inferior leads, and persistence of atrial septal defect.
***Contra Indications for TS Access:*** While the relative contraindications are anatomical abnormalities, abnormal coagulation and history embolization, the main contraindication is presence of atrial thrombus or mass. Although the organized thrombus may not be problematic for TS, it may not be properly predicted in advance whether the thrombus is fresh or organized. The coumadine could be prescibed to resolve the thromnbus prior TS procedure [11,16].

## 5 Current solutions for difficult puncture

This section details the solutions that may be adopted for difficult punctures.

***Needle wire SafeSept:*** The NiTi guidewire, which is sharp and has J-shaped tip is inserted into the needle. Upon the puncture and passage of NiTi guidewire through FO immediately takes the J shaped inside the LA. Given the presence of j-shaped guidewire inside the LA, the entrance of needle and sheath inside LA will be safe, i.e., no perforation inside LA may happen, as they advance over the SafeSept wire.
***RF system:*** It applies 2-second of RF energy to create a hole at Septum. The pros of RF method is that the catheter's distal end is more flexible than a needle and that is helpful when specific crossing site is needed. Moreover, the excessive force and so the risk of needle jumping inside the LA reduces. However, the cons of RF method are that it has not yet established if it has the same safety profile as a needle when cardiac wall perforation occurs [17].

## 6 Conclusions

To realize the development of TMVR systems for higher surgical risk patients still require in-depth understanding of many anatomic, procedure and biomedical engineering constraints. To make TMVR competitive to TAVI, efforts are being made to address the challenges of D shaped annulus, non- existent fibrosis and calcified structure and complex sub valvular apparatus. TMVR anchoring systems are broadly progressing in the realms of tether, new leaf engagement and mitral annulus clamping. Similarly, transseptal chordal devices and access modalities exemplify leading solutions to diversify solution set for responsive patients with MR. The current bottlenecks with transseptal access include; aneorysmal atrial septum, distorted anatomy, thrombosis and contra indications. Furthermore, in hand solutions for difficult puncture scenarios is briefly elaborated.

## Declaration of Competing Interest

The authors declare that they have no known competing financial interests or personal relationships that could have appeared to influence the work reported in this paper.
